# Molecular characterization of a novel chitinase *Cm*Chi1 from *Chitinolyticbacter meiyuanensis* SYBC-H1 and its use in *N*-acetyl-d-glucosamine production

**DOI:** 10.1186/s13068-018-1169-x

**Published:** 2018-06-26

**Authors:** Alei Zhang, Yumei He, Guoguang Wei, Jie Zhou, Weiliang Dong, Kequan Chen, Pingkai Ouyang

**Affiliations:** 1 College of Biotechnology and Pharmaceutical Engineering, NanjingTech University, Nanjing, 211800 People’s Republic of China; 2 State Key Laboratory of Materials-Oriented Chemical Engineering, NanjingTech University, Nanjing, 211800 People’s Republic of China

**Keywords:** *Chitinolyticbacter meiyuanensis* SYBC-H1, Multi-functional, Chitinase, Colloidal chitin, *N*-acetyl-d-glucosamine

## Abstract

**Background:**

*N*-acetyl-d-glucosamine (GlcNAc) possesses many bioactivities that have been used widely in many fields. The enzymatic production of GlcNAc is eco-friendly, with high yields and a mild production process compared with the traditional chemical process. Therefore, it is crucial to discover a better chitinase for GlcNAc production from chitin.

**Results:**

A novel chitinase gene (*Cmchi1*) cloned from *Chitinolyticbacter meiyuanensis* SYBC-H1 and expressed in *Escherichia coli* BL21(DE3) cells. The recombinant enzyme (*Cm*Chi1) contains a glycosyl hydrolase family 18 catalytic module that shows low identity (12–27%) with the corresponding domain of the well-characterized chitinases. *Cm*Chi1 was purified with a recovery yield of 89% by colloidal chitin affinity chromatography, whereupon it had a specific activity of up to 15.3 U/mg. *Cm*Chi1 had an approximate molecular mass of 70 kDa after the sodium dodecyl sulfate-polyacrylamide gel electrophoresis, and its optimum activity for colloidal chitin (CC) hydrolysis occurred at pH 5.2 and 50 °C. Furthermore, *Cm*Chi1 exhibited *k*_cat_/*K*_m_ values of 7.8 ± 0.11 mL/s/mg and 239.1 ± 2.6 mL/s/μmol toward CC and 4-nitrophenol *N,N′*-diacetyl-β-d-chitobioside [*p*-NP-(GlcNAc)_2_], respectively. Analysis of the hydrolysis products revealed that *Cm*Chi1 exhibits exo-acting, endo-acting and *N*-acetyl-β-d-glucosaminidase activities toward *N*-acetyl chitooligosaccharides (*N*-acetyl CHOS) and CC substrates, behavior that makes it different from typical reported chitinases. As a result, GlcNAc could be produced by hydrolyzing CC using recombinant *Cm*Chi1 alone with a yield of nearly 100% and separated simply from the hydrolysate with a high purity of 98%.

**Conclusion:**

The hydrolytic properties and good environmental adaptions indicate that *Cm*Chi1 has excellent potential in commercial GlcNAc production. This is the first report on exo-acting, endo-acting and *N*-acetyl-β-d-glucosaminidase activities from *Chitinolyticbacter* species.

**Electronic supplementary material:**

The online version of this article (10.1186/s13068-018-1169-x) contains supplementary material, which is available to authorized users.

## Background

Chitin is the second most abundant polysaccharide in nature after cellulose, and it comprises 20–30% of the shells of crustaceans [1]. About 6–8 million tonnes of crab, shrimp and lobster shell wastes are produced globally annually. This causes severe environmental pollution because it is not utilized effectively [2]. *N*-acetyl-d-glucosamine (GlcNAc), the monomeric unit of the polymer chitin, exhibits many bioactivities that have been used widely in many fields, such as the food, pharmaceutical, biomedical and fine chemicals industries [3]. Therefore, it would be economically and environmentally significant if a way could be found to efficiently produce GlcNAc from normally discarded chitin resources.

Production methods that convert waste chitin to GlcNAc have been widely explored to date [4–6]. Generally, GlcNAc has been commercially produced by the acid hydrolysis of chitin at high temperature and at high concentrations [3]. However, this method has some disadvantages, including high cost, low yield and the environmental pollution it generates, which limits its application in the food industry [7]. In recent years, researchers have paid more attention to the enzymatic production of GlcNAc because this approach is eco-friendly, with high yields and a mild production process [3]. Hence, chitin-degrading enzymes are used for the production of GlcNAc on an industrial scale.

Chitinases are a group of enzymes (EC.3.2.14) that include endochitinase [hydrolyzes chitin to *N*-acetyl chitooligosaccharides (CHOS)], exochitinase (hydrolyzes chitin to [GlcNAc]_2_) and *N*-acetyl-β-d-glucosaminidases (hydrolyze [GlcNAc]_2_ or *N*-acetyl CHOSs to GlcNAc) [8]. Chitinases can be secreted by some microorganisms, insects, higher plants and animals, in which they play a significant physiological role depending on their origin [8].

Chitinases derived from bacteria have potential for industrial application due to their excellent properties and ease of cultivation [9]. The crude enzymes from *Aeromonas hydrophila* H-2330 were used to produce GlcNAc from chitin with a yield of 77% [10]. A novel combination of bacterial chitinases from *Serratia marcescens* and insect chitinolytic enzymes was developed to efficiently produce GlcNAc with a yield of 1.4 from 10 g/L mycelial powder [11]. However, there are accumulations of oligomers (mainly dimer) during these processes, which increase the difficulty of separation of the desired product and hinder their application in industrial scale production. To enhance the yield of GlcNAc, a multienzyme system consisting of a chitinase and an *N*-acetyl-β-d-glucosaminidase is urgently needed, but such a system would increase the cost [12]. Therefore, finding a multi-functional chitinase is crucial for the enzymatic industrial production of GlcNAc.

Strain SYBC-H1, with an excellent capability of degrading chitin, was isolated and classified as a novel genus belonging to the family *Neisseriaceae* by Hao et al. [13]. The fermentative production of extracellular chitinases from strain SYBC-H1 was enhanced by optimization of culture condition and medium [14]. In previous studies, SYBC-H1 chitinases production was also optimized using a staged pH control strategy [15], and GlcNAc was produced from crude chitin powders using the chitinases from strain SYBC-H1 [16]. However, there are few reports about the coding genes, cloning, enzyme characteristics and catalytic characteristics of the chitinases from the family *Neisseriaceae* at present [17, 18]. Research about bacterial chitinases has mainly focused on *S. marcescens*, *A. hydrophila*, *Bacillus subtilis and Bacillus licheniformis* [10].

In this study, a gene encoding a multi-functional chitinase (*Cm*Chi1) from strain SYBC-H1 was cloned, based on the results of peptide mass fingerprinting, and heterologously expressed in *Escherichia coli* BL21(DE3). Analysis of its sequence and enzymatic properties and kinetics revealed that the multi-functional chitinase (*Cm*Chi1) is a novel GH18 member with substrate specificity toward CC, *N*-acetyl CHOS and *p*-NP-(GlcNAc)_2_. Furthermore, the enzymatic production and purification of GlcNAc from CC was also investigated using the purified *Cm*Chi1.

## Methods

### Strains, plasmids and chemicals

*Chitinolyticbacter meiyuanensis* SYBC-H1, which produces extracellular chitinases with high activity, was provided by Hao et al. [13]. Strain SYBC-H1 was cultivated in a culture medium (pH 7.0) consisting of 4.0 g/L glucose, 4.0 g/L peptone, 4.0 g/L yeast extract, 0.7 g/L KH_2_PO_4_, 0.3 g/L K_2_HPO_4_ and 0.5 g/L MgSO_4_ at 37 °C for 12 h on a rotary shaker (200 rpm). The pET-28a(+) plasmid was used as the expression vector for the overexpression of the chitinase gene in *E. coli* BL21(DE3) (Novagen Co., Shanghai, China), which was cultivated in Luria–Bertani (LB) broth or on agar plates containing 50 μg/mL kanamycin and 20 g/L colloidal chitin.

The gene (*Cmchi1*) was amplified from the genomic DNA of strain SYBC-H1 using the primers listed in Table 1 with PrimeSTAR high sensitivity (HS) DNA polymerase. The molecular reagents were purchased from Takara Bio Inc. (Dalian, China). All chemicals used in this study were of analytical grade or higher purity. Colloidal chitin (CC) was prepared as described by Gao et al. [19].

**Table 1:** Oligonucleotide primers used for PCR

Primer	Sequence (5ʹ-3ʹ)	Design basis	Position in *Cmchi1* gene (5ʹ-3ʹ)
*Cmchi1*PF	GCTGCCACCCCGACACCGGTTTCGGCTACC	Peptide fragments 1	253–282
*Cmchi1*PR	TGCGTTCTTCAGCACCTTGTAGTCCTC	Peptide fragments 2	1744–1770
ARB1	GGCCACGCGTCGACTAGTACNNNNNNNNNNGATAT	Arbitrary primer for TAIL-PCR	None
ARB2	GGCCACGCGTCGACTAGTACNNNNNNNNNNACGCC	Arbitrary primer for TAIL-PCR	None
ARB3	GGCCACGCGTCGACTAGTAC	Arbitrary primer for TAIL-PCR	None
F-SP1	GGCGCCGCTCGCGGCACCTAC	Specific primer for TAIL-PCR	1711–1731
F-SP2	GGTCGTGGCTGGACTGGCGTGCCG	Specific primer for TAIL-PCR	1654–1677
R-SP1	GTCGTCGCCACCCCGACACCG	Specific primer for TAIL-PCR	292–312
R-SP2	CGGTGTCGGGGTGGCGACGAC	Specific primer for TAIL-PCR	344–366
*Cmchi1*-F	CATGCCATGGATGTCGCAAATCAATCGCTTC	Forward primer for *CmChi1*	1–21
*Cmchi1*-R	CCGCTCGAGTTACTTGTTCATGTTGCCCATG	Reverse primer for *CmChi1*	1950–1977

### Purification and identification of the chitinase from *C. meiyuanensis* SYBC-H1

Cells of strain SYBC-H1 were cultured at 37 °C and the supernatant was collected as crude enzyme by centrifugation (5810R Eppendorf, Ltd., Shanghai, China) at 5000×*g* at 4 °C. The enzyme was purified by chitinase–glycogen complex precipitation followed by autodigestion of the complex. A total of 1% (w/v) CC as adsorbent material was added to the crude enzyme at a ratio of 1:2 and incubated for 5 min in an ice bath. Subsequently, the supernatant was removed by centrifugation at 8000×*g* at 4 °C for 1 min. The impurity proteins in the precipitate were eluted three times using 1 M NaCl and then salt ions were removed by precooled ultrapure water. Finally, the chitinase–glycogen complex was re-suspended and incubated at 40 °C for 4 h to hydrolyze CC to a reducing sugar, which was removed by dialysis and ultrafiltration. The purified chitinase was analyzed by native-polyacrylamide gel electrophoresis (native-PAGE) using 8% acrylamide, according to the method described by Laemmli [20].

After native electrophoresis, the gel was sliced vertically into two parts. One part was stained with 0.1% Coomassie brilliant blue R-250 to determine the protein purity, and the other part was incubated in 50 mM sodium citrate buffer (pH 5.2) containing 0.5 mM 4-methylumbelliferyl *N,N′*-diacetyl-β-d-chitobioside (4-MU-[GlcNAc]_2_) at 37 °C for 30 min. The proteins containing chitinase became visible as fluorescent at 340 nm. Then, the stained gels were compared with the zymogram to determine the position of the chitinase. The corresponding proteins in the native PAGE gel were sliced for peptide fingerprint analysis using the electrospray ionization quadrupole time-of-flight mass spectrometer (ESI-Q-TOF MS/MS) technique (PROTTECH, Inc., Suzhou, China). These masses were then compared to theoretical mass values in the Mascot website databases (http://www.matrixscience.com) to reveal the amino acid sequences of the peptide fragments.

### Molecular cloning and sequence analysis of *Cmchi1*

Genomic DNA was extracted from strain SYBC-H1 cells using the TIANamp Bacteria DNA Kit (Tiangen Biotech Co., Ltd., Beijing, China). Primers *Cmchi1*PF and *Cmchi1*PR, which were designed based on two peptide fragments of *Cm*Chi1 (NH_2_-AATPTPVSAT and NH_2_-EDYKVLKNA), were applied to amplify the internal 1518 bp of *Cmchi1*. The 5′-nucleotide sequence and 3′-nucleotide sequence were amplified by thermal asymmetric interlaced (TAIL)-PCR using the specific primers F-SP2 and F-SP1, and R-SP2 and R-SP1, and the arbitrary primers ARB1, ARB2 and ARB3, as described previously [21]. The complete gene was PCR-amplified using the following primers: *Cmchi1*-F containing an *Nco*I site at the initiation site of the *Cmchi1* gene; and *Cmchi1*-R containing an *Xho*I site after the end of the *Cmchi1* gene and deletion of the stop codon.

Nucleotide and amino acid sequences were analyzed using Snap Gene™ 1.1.3 software (http://www.snapgene.com/) and the ExPASy Protparam tool (http://web.expasy.org/protparam/). The DNA and protein sequence alignments were performed via the NCBI server with the programs BLASTN and BLASTP (http://blast.ncbi.nlm.nih.gov/Blast.cgi), respectively. The conserved domains and the GH family classification were identified via the website (http://prosite.expasy.org/scanprosite/) and aligned using the Clustalx program (version 1.81). Signal peptide was predicted in the SignalP 4.1 server (http://www.cbs.dtu.dk/services/SignalP/).

### Expression of *Cmchi1* and purification of *Cm*Chi1

The PCR products as described earlier were digested using *Nco*I and *Xho*I and inserted into the *Nco*I–*Xho*I sites of pET-28a(+) expression plasmid with an C-terminal His_6_-tag to obtain the recombinant plasmid, which was then transformed into *E. coli* BL21(DE3). The transformed cells were grown in a 1.4-L INFORS HT Multifors fermenter (Infors Biotechnology Co., Ltd., Beijing, China) containing 1 L LB medium and 50 μg/mL kanamycin at 37 °C, with an aeration ratio of 1 vvm (vessel volume per minute) and agitation speed of 250 rpm, until the optical density at 600 nm (OD_600_) reached 0.6–0.8. The recombinant *Cm*Chi1 was induced at a final concentration of 0.2 mM isopropyl-β-d-thiogalactopyranoside (IPTG) at 25 °C for 8 h.

The cells were harvested, washed and resuspended in an equilibration buffer [50 mM phosphate buffer saline (PBS), pH 7.0] at 4 °C and lysed by JY92-IIN ultrasonication (Ningbo xingzhi biotechnology, Ltd., Ningbo, China). The lysate was centrifuged at 12,580×*g* for 20 min, and the supernatant was used as a crude enzyme solution. The recombinant *Cm*Chi1 were purified using a fast protein liquid chromatography (FPLC) system (GE AKTA Pure 150; General Electric Co., Fairfield, America) with a Ni-nitrilotriacetic acid affinity chromatography (Ni–NTA) column (His Trap™ FF5 mL) according to the manufacturer’s instructions. In addition, the method of chitinase–glycogen complex precipitation above was also used for *Cm*Chi1 purification.

Activity screening against the various substrates was performed by 3, 5-dinitrosalicylic acid (DNS) assays [22]. Unless otherwise indicated, the enzyme reaction mixture containing the suitably diluted enzyme and different polysaccharide substrates at a final concentration of 10 g/L in 50 mM sodium citrate buffer (pH 5.2) was incubated for 30 min at 50 °C. The amount of reducing sugars was determined spectrophotometrically at 540 nm [15]. One unit of chitinase activity was defined as the amount of enzyme required to produce 1 μmol reducing sugar at 50 °C in 1 min. All chitinase activities were assayed in triplicate and the average enzyme activity with standard deviation was calculated. Protein concentrations were determined at 280 nm using the Bradford method [23] with bovine serum albumin as the standard. All protein samples were analyzed by reductive SDS-PAGE with 20 mM β-mercaptoethanol incubation. A premixed protein marker (Takara Biotechnology Co., Ltd., Nanjing, China) containing 180-, 140-, 100-, 75-, 60- and 45-kDa proteins was used as the molecular mass standard.

### Enzymatic characterization

With 10 g/L CC as the substrate, the optimal temperature for the chitinase activity was determined over the range of 25–60 °C in 50 mM sodium citrate buffer (pH 5.2). Enzyme thermostability was determined by measuring the residual activity after pre-incubation of the purified enzyme in 50 mM sodium citrate buffer (pH 5.2) at 25–60 °C without substrate for 2 h. The optimal pH for the chitinase activity was assessed in several buffers at 45 °C. The following buffers were used: 50 mM sodium citrate buffer, pH 3.6–6.2; 50 mM PBS buffer, pH 5.6–8.4; and 50 mM 2-(cyclohexylamino)-1-ethanesulfonic acid (CHES) buffer, pH 8.0–10.0. To measure the pH stability, the enzyme was incubated at 4 °C for 2 h in the different buffers and the residual activities were determined against 10 g/L CC.

The effects of metal ions on the activity were also determined in this study. Purified *Cm*Chi1 was treated with 10 mM EDTA for 5 h at 4 °C and then dialyzed against 50 mM sodium citrate buffer (pH 5.2) to remove the EDTA. The activities were assayed as described previously and compared to the activity of an untreated enzyme solution incubated under the same conditions. For reactivation, the metal-free enzyme was incubated with metal ions (Ca^2+^, Cu^2+^, Co^2+^, K^+^, Na^+^, Al^3+^, Ba^2+^, Ni^2+^, Zn^2+^, Mg^2+^, Mn^2+^, Ag^+^, and Fe^2+^) at a final concentration of 10 mM for 10 min, and the remaining activity was determined. The activity prior to EDTA treatment was used as the control (100%).

The substrate specificity was determined with various 1% (w/v) carbohydrates including polysaccharides [CC, chitin powder, carboxymethyl cellulose (CMC), hemicellulose powder, amylose powder, chitosan powder] and *N*-acetyl CHOSs [Degree of polymerization (DP), 2–6] as substrates under the optimum conditions. The amount of reducing sugars released from these polysaccharides substrates was estimated by the DNS method as described previously while the amount of reducing sugars from *N*-acetyl CHOSs was quantified with HPLC. The enzymatic activities for 4-nitrophenol *N,N′*-diacetyl-β-d-GlcNAc (*p*-NP-GlcNAc) and *p*-NP-(GlcNAc)_2_ were determined by measuring the amounts of *p*-NP released. Chitinase assay with *p*-NP-GlcNAc and *p*-NP-(GlcNAc)_2_ as the substrate: a total of 20 μL of the enzyme solution were added to 0.98 mL of 0.25 mmol/L (*p*-NP-GlcNAc) and *p*-NP-(GlcNAc)_2_ in 50 mM sodium phosphate buffer (pH 7.0) and incubated at 50 °C for 10 min. The reaction was terminated by adding 2 mL of NaOH (0.5 mol/L). One unit of chitinase activity was defined as the amount of enzyme required to release 1 μmol *p*-NP from the substrate per minute at 50 °C.

The apparent kinetic parameters against CC and *p*-NP-(GlcNAc)_2_ under the optimal conditions for *Cm*Chi1 were measured, respectively. For CC, the initial velocities were determined by incubating 36 ug purified *Cm*Chi1 with CC concentrations ranging from 1 to 15 mg/mL at 50 °C in 1 mL reaction system (50 mM sodium citrate buffer, pH 5.2) for 20 min. The amount of reducing sugars released from these CC was estimated by the DNS method as described previously. For *p*-NP-(GlcNAc)_2_, the initial velocities were determined by incubating 3 ug purified *Cm*Chi1 with *p*-NP-(GlcNAc)_2_ concentrations ranging from 0.05 to 1.0 μmol/mL at 50 °C in 1 mL reaction system (50 mM sodium citrate buffer, pH 5.2) for 5 min. The reaction was terminated by adding 1 mL of NaOH (0.5 M).

The *K*_m_ and *V*_max_ values were obtained by Lineweaver–Burk plots [24], when the reaction rate of *Cm*Chi1 were linearly with concentration of *p*-NP-(GlcNAc)_2_ (0.05–0.4 μmol/mL) and CC (1–4 mg/mL).

### Affinity of the purified *Cm*Chi1 for various polysaccharides

The binding of *Cm*Chi1 to various polysaccharides was investigated in 2-mL mixtures containing 150 μg *Cm*Chi1 and 1 g/L CC, chitin powder, chitosan powder, CMC in 50 mM sodium citrate buffer (pH 5.2) with 0.5 M NaCl. The binding mixture was incubated for 60 min at 4 °C with rotary shaking at 1000 r/min and then centrifuged at 12,000×*g* for 2 min at 4 °C at intervals of 5–60 min. The protein concentration in the supernatant was measured by the Bradford method as described previously. The amount of protein adsorbed was equal to the difference between the total protein concentration and the protein concentration in the supernatant. All experiments were performed in triplicate.

### Detection of hydrolysis products

Reaction mixtures containing purified *Cm*Chi1 (50 μg) and various substrates [CC and *N*-acetyl CHOSs (DP 2–6)] at a final concentration of 10 g/L were incubated in 100 mL of 50 mM sodium citrate buffer (pH 5.2) at 50 °C for various time intervals. In each case, the supernatant after hydrolysis was diluted with ethanol (200 mL) and centrifuged at 8000×*g* for 10 min to remove the protein. The collected supernatant was concentrated using a rotary evaporator and then dropped into absolute ethanol (100 mL) with stirring to form crystals, which were left in a refrigerator at 4 °C overnight to complete the precipitation. The precipitate was separated by centrifugation at 2000×*g* and dried under vacuum for 24 h.

Products were detected by an Agilent 1260 series LC system (Agilent Technologies, Santa Clara, USA) with a UV detector at 210 nm using a separation column (Alltech chrom Prevail Carbohydrate ES 5 μ column (4.6 mm × 250 mm), Santa Clara, USA). The mobile phase was composed of acetonitrile:water and the gradient elution conditions were: 0 min, 75% acetonitrile; 7 min, 75% acetonitrile; 8 min, 65% acetonitrile; 15 min, 65% acetonitrile; 16 min, 75% acetonitrile; 22 min, 75% acetonitrile. The GlcNAc yield from CC was calculated according to the following equation:

$${\text{GlcNAc yield}}\left( \% \right) = {\text{GlcNAc}}\left( {\text{g}} \right)/{\text{CC}}\left( {\text{g}} \right).$$

The mass spectrum of GlcNAc was provided by Biopharmpt Co., Ltd. (Nanjing, China).

### Nucleotide sequence accession number

The sequence of the chitinase gene *Cmchi1* has been deposited in the GenBank database under the accession number MG210568.

## Results and discussion

### Purification and identification of chitinases from strain SYBC-H1

**Fig. 1:**
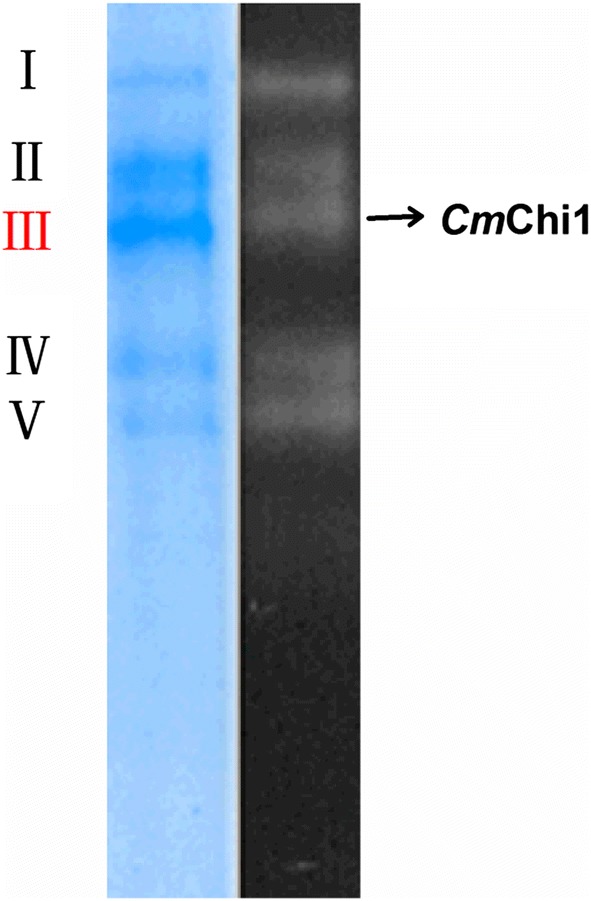
The native PAGE and zymogram analysis of all chitinases purified from *C. meiyuanensis* SYBC-H1 cells using chitinase–glycogen complex precipitation method. The amount of protein applied to the gel is 10 μg. The gel slice on the left is the coomassie stained one and that on the right is the zymogram. Five proteins [I, II, III (*Cm*Chi1), IV and V] with chitinase activity were purified using chitinase–glycogen complex precipitation followed by autodigestion of the complex

A total of five proteins (designated protein I, II, III, IV and V) with chitinase activity from supernatant of strain SYBC-H1 were obtained, as shown by the zymogram (Fig. 1). Affinity purification of chitinase from crude extract, followed by autodigestion, a 38.3-fold increase in enzyme specific activity was obtained.

The stained gel proteins were excised and analyzed with matrix-assisted laser desorption ionization–time-of-flight peptide mass fingerprinting (PMF), and the results of PMF were interpreted by referencing the Mascot database [25]. Proteins receiving the highest molecular weight search scores were selected as the peptide fragments of chitinases. Surprisingly, the results of PMF from protein I, II, IV and V did not match the existing amino acid sequences in the database, indicating these chitinases have novel amino acid sequences. Peptide fragments of protein III were detected with the amino acid sequences of AATPTPVSAT and EDYKVLKNA and LTFINYAFGNVYQK, which shared high identity to the reported peptides of chitinases from *Lactobacillus* sp. wkB8 (71%, WP_051083235) [26], *Chitiniphilus shinanonensis* (100%, BAK53931) [18] and *Ardenticatena maritima* (88%, GAP63111) [27].

### Cloning of the chitinase gene and sequence analysis

The amino acid sequences of these peptide fragments (NH_2_-AATPTPVSAT and NH_2_- EDYKVLKNA) were used to design primers to amplify *Cmchi1* from the genomic DNA of *C. meiyuanensis* SYBC-H1. The degenerate primers *Cmchi1*PF and *Cmchi1*PR were used to amplify the internal 1518-bp fragment of *Cmchi1*. The 5ʹ and 3ʹ terminal nucleotide sequences of *Cmchi1* were amplified by TAIL-PCR. Based on the 5ʹ and 3ʹ information obtained from these reactions, oligonucleotides for the complete nucleotide sequence were designed, which were then PCR-amplified using primers *Cmchi1*-F and *Cmchi1*-R. Finally, a 1977-bp fragment of *Cmchi1* was obtained, which encoded a protein of 658 amino acids with a calculated molecular mass and pI of 70.1 kDa and 7.2, respectively. Domain structure prediction analysis indicated that *Cm*Chi1 possesses a single glycoside hydrolase family 18 catalytic domain (residues 237–643), a predicted N-terminal signal peptide (residue 23) and two carbohydrate binding domains (residues 27–73 and 123–165). The reported chitinases usually possess at least one carbohydrate binding domain according to the literatures [12, 18, 28]. Chitinases with multiple chitin binding domains may have stronger affinity than that with a single binding domain, which can explain the reason why the *Cm*Chi1 has a strong affinity for CC.

**Fig. 2:**
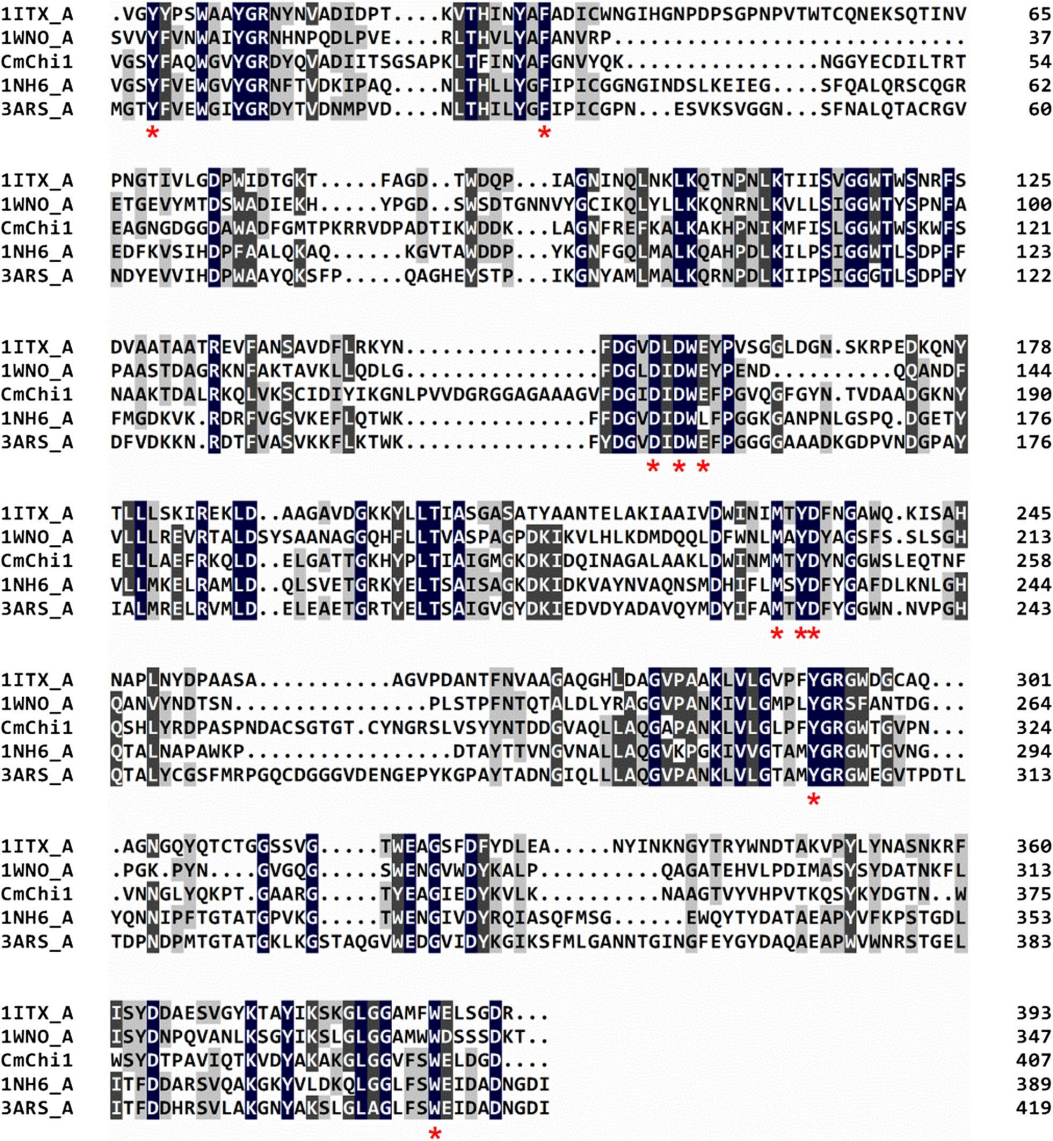
Multiple alignments of the amino acid sequences of GH18 catalytic domain in *Cm*Chi1 and related GH18 family chitinases. The other listed sequences included the chitinases from *Bacillus circulans* Wl-12 (PDB No. 1ITX), *Serratia marcescens* ATCC990 (PDB No.1NH6), *Vibrio harveyi* (PDB No. 3ARS) and *Aspergillus fumigatus* Yj-407 (PDB No. 1WNO). Gray represents conserved residues between sequences. The red asterisks represent the conserved catalytic active sites. The numbers on the right are the positions of the first amino acid residue in the whole sequence

The BLASTP analysis showed that *Cm*Chi1 shares the highest identity (99%) with a chitinase (AGC59908.1) belonging to the GH18 family from *Staphylococcus* sp. J2, followed by 85% identity with the chitinases from *C. shinanonensis* (BAK53886.1) [18], *Chitinibacter* sp. ZOR0017 (WP_081986537.1) and *Chitinibacter tainanensis* (WP_084414662.1). However, none of these proteins has been characterized. The amino acid sequences of *Cm*Chi1 were searched against the PDB database, and the results showed 33–37% identity with proteins from the GH18 family, such as chitinase A1 (1ITX) from *Bacillus circulans* Wl-12, chitinase A (1NH6) from *S. marcescens* ATCC990, and chitinase (1WNO) from *Aspergillus fumigatus* Yj-407 [29]. Multiple alignments of the deduced GH18 domain amino acid sequences of *Cm*Chi1 and other chitinases indicated the active sites of *Cm*Chi1 consist of Y-240, F-272, D-402, D-404, E-406, M-479, Y-481, D-482, Y-551 and W-638, which are highly conserved among GH18 members (Fig. 2).

### Expression of *Cmchi1* and purification of recombinant *Cm*Chi1

**Fig. 3:**
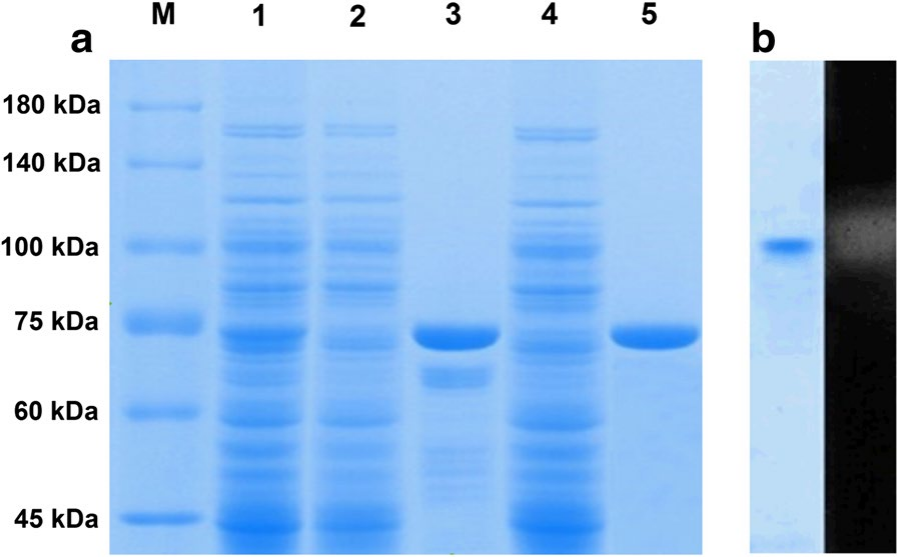
Polyacrylamide gel electrophoresis (PAGE) analysis of chitinases from *C. meiyuanensis* SYBC-H1 and recombinant *E. coli* BL21(DE3). **a** SDS-PAGE analysis of the expression and purification of recombinant *Cm*Chi1. The amount of protein applied to the gel is 10 μg *Lane* M, protein molecular mass markers; *lane 1*, total protein of recombinant *Escherichia coli* BL21(DE3); *lane 2*, extract of a 50-mM imidazole eluted sample from a Ni–NTA column; *lane 3*, extract of a 200-mM imidazole eluted sample from a Ni–NTA column; *lane 4*, eluted sample using 1 M NaCl from the complex containing CC and *Cm*Chi1; *lane 5*, digestion extract without reducing sugar from the complex containing CC and *Cm*Chi1. **b** Zymogram analysis of purified recombinant *Cm*Chi1 by a native PAGE. The left gel slice is the coomassie stained one and that on the right is the zymogram. The zymogram assay was conducted in 50 mM sodium citrate buffer (pH 5.2) containing 0.5 mM 4-methylumbelliferyl *N,N′*-diacetyl-β-d-chitobioside (4-MU-[GlcNAc]_2_) at 37 °C for 30 min, then placed at 340 nm for showing fluorescence

*Cmchi1* with and without the signal peptide sequence were successfully overexpressed in *E. coli* BL21(DE3) and showed no alteration on activity and protein secretion (data not shown). Thus, the *Cm*Chi1 with the signal peptide was used in next experiments. The produced and purified recombinant *Cm*Chi1 was analyzed with SDS-PAGE. The lane 1 in Fig. 3a showed that the localization of the *Cm*Chi1 was in supernatant of the cell-free extract of the recombinant *E.coli* BL21(DE3)-(pET28a(+)-*Cmchi1*), indicated the *Cm*Chi1 with the signal peptide was soluble. The molecular weight of *Cm*Chi1 is approximately 70 kDa on SDS-PAGE, which is in agreement with 70.9 kDa calculated from the amino acid sequence containing the signal peptide and the His-tag (Fig. 3a). The recombinant *Cm*Chi1 was eluted with 200 mM imidazole from a Ni–NTA resin with a recovery yield of 70.3% (Table 2). Although the recovery yield was higher than other reported data for chitinase purification, about 30% of the target protein was still lost during the purification process [12]. Therefore, the chitinase–glycogen complex precipitation method as described in “Methods” was also applied in the purification of recombinant *Cm*Chi1. The outstanding feature of this method is that the recombinant *Cm*Chi1 could be efficiently and rapidly absorbed (≤ 5 min) with a specific activity and recovery yield of 15.3 U/mg and 89.0%, respectively (Table 2). Meanwhile, a single protein was exhibited on the SDS-PAGE gel (Fig. 3a), which indicated chitinase–glycogen complex precipitation is a better method for *Cm*Chi1 purification. Furthermore, the chitinase activity of the purified *Cm*Chi1 was validated with native PAGE and zymogram analysis (Fig. 3b).

**Table 2:** Purification of recombinant *Cm*Chi1 by different methods

Purification method	Total activity (U)	Total protein (mg)	Specific activity (U/mg)^a^	Purification (fold)	Recovery yield (%)
Crude enzyme	198.6	521.1	0.4	0	100
Ni–NTA resin	139.6	12.3	11.4	29.9	70.3
Chitinase–glycogen complex precipitation	176.8	11.6	15.3	40.2	89.0

^a^Enzymatic activity was measured in 50 mM citrate buffer (pH 5.2) at 50 °C for 30 min using 1% (w/v) CC as the substrate

Ueda and Kurosawa reported that the chitinase purified from *Paenibacillus thermoaerophilus* TC22-2b using CC adsorption possessed a specific activity of 1.3 U/mg and recovery yield of 27.4%, which were both much lower than those obtained in this study [30]. Skujins et al. also reported the adsorption of *Streptomyces* sp. chitinase on chitin in universal buffer was fast and completed within 2 min [31].

*Cm*Chi1 exhibited an activity of 15.3 U/mg toward CC, which is comparable to that of the chitinase (19.9 U/mg) from *Paenibacillus barengoltzii* [12] and higher than the activities of most reported chitinases from *Chitiniphilus shinanonensis* SAY3^T^ (3.8 U/mg) [32], *Bacillus* sp. WY22 (3.7 U/mg) [33]*, P. thermoaerophilus* TC22-2b (1.3 U/mg) [30] and *Bacillus* sp. DAU101 (0.73 U/mg) [34].

### Affinity of purified *Cm*Chi1 for polysaccharides

**Fig. 4:**
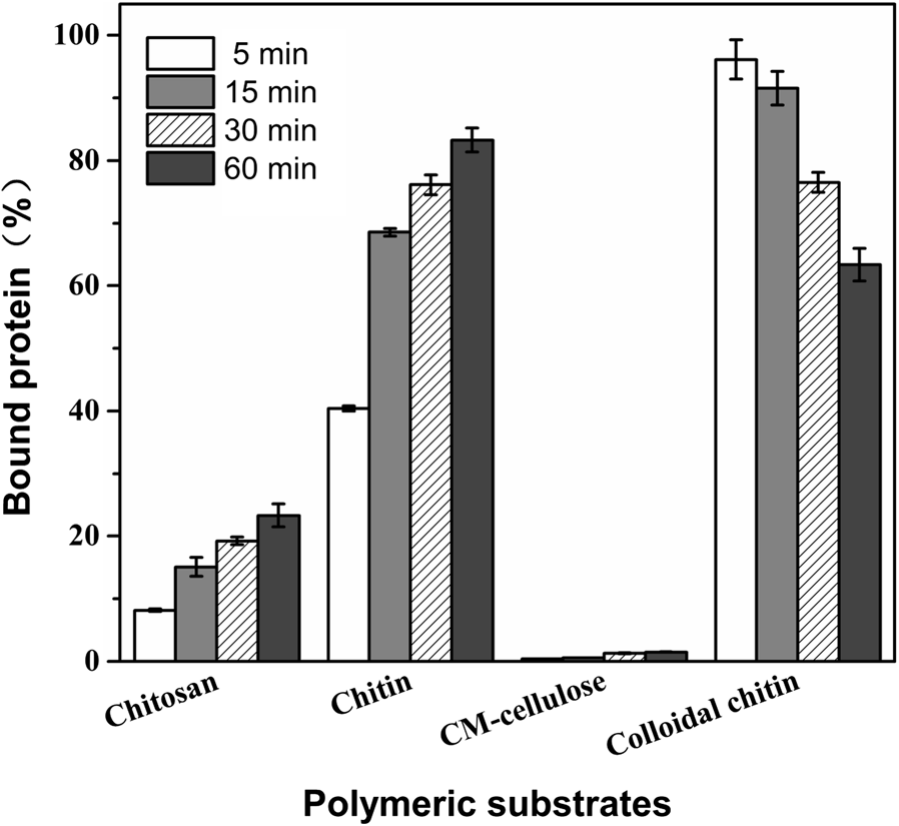
Affinity of the purified *Cm*Chi1 for polysaccharides. The reaction mixture of 2-mL contained 150 μg of *Cm*Chi1 and 1 g/L of one of the polymeric substrates in 50 mM sodium citrate buffer couple with 0.5 M NaCl (pH 5.2). The affinity mixture was incubated at 4 °C with rotary shaking at 1000 rpm. Samples from different time intervals were immediately centrifuged, and the unabsorbed protein in the supernatant was measured. The adsorbed protein was calculated as the amount of protein in the control minus that in the supernatant. All experiments were performed in triplicate

The chitinase–glycogen complex precipitation method is very effective for chitinase purification, thus the affinity of the purified *Cm*Chi1 for polysaccharides was determined in this study. The amount of protein bound to chitin powder and chitosan powder increased with increasing time, and reached 83.3 and 23.3% after 1 h, respectively (Fig. 4). However, the bound protein on CC rapidly reached a maximum amount (96.2%) after 5 min and then decreased gradually due to the hydrolysis of CC by the bound protein over time (Fig. 4). These results are in agreement with our previous study, which also showed that the ability of the chitinase to adsorb on CC was greater than its ability to adsorb on chitin powder over a short time period. We explained the reason for this by comparing the surface morphologies using scanning electron microscopy (SEM) [16]. Purushotham and Podile also reported that the affinity of chitinase *Sp*ChiD from *Serratia proteamaculans* 568 reach 80% toward CC, which is lower than *Cm*Chi1 [35]. In addition, *Cm*Chi1 exhibited little affinity toward CM-cellulose (CMC), whereas different chitinases from *Enterobacter* sp. NRG4 A revealed a substrate-binding capacity of 15.2% for CMC [36]. These results indicated *Cm*Chi1 has a specific substrate binding mechanism for polysaccharides.

### Effects of temperature, pH and metal ions on the enzymatic activity and stability

**Fig. 5:**
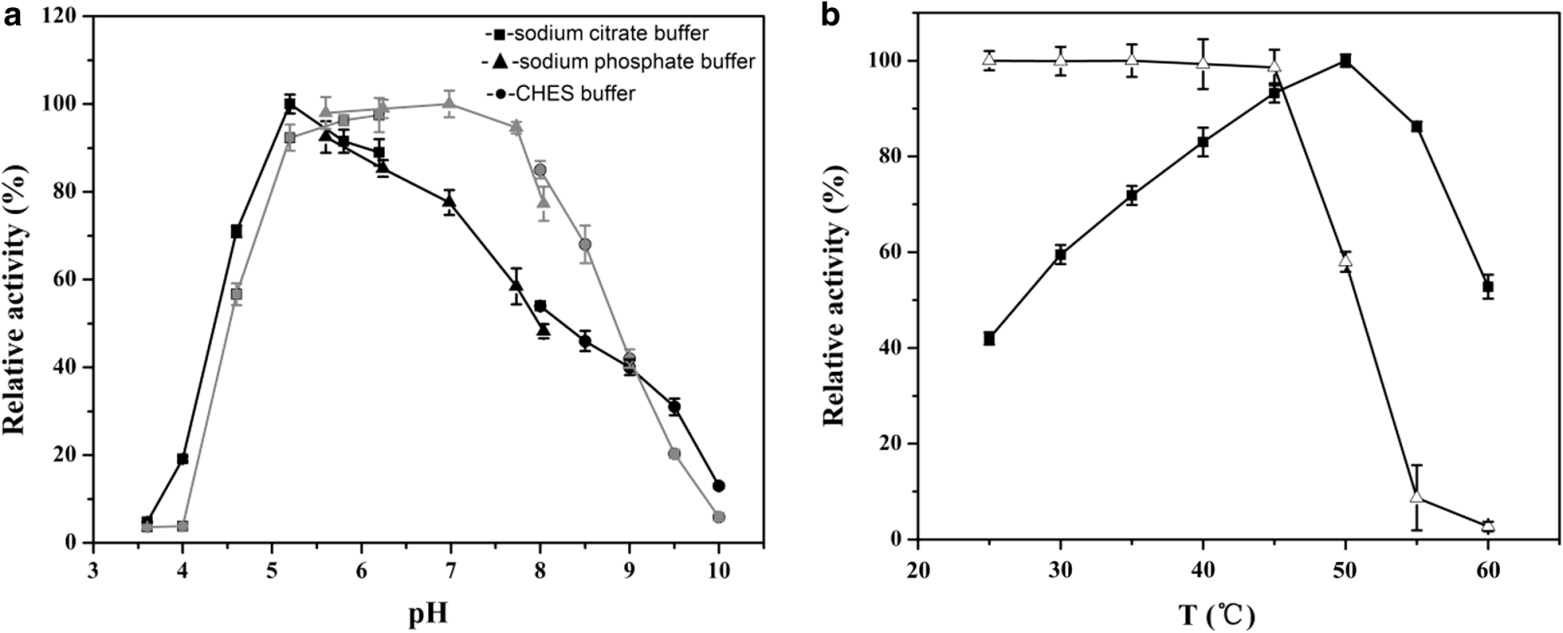
Effect of pH and temperature on the activity and stability of *Cm*Chi1. **a** Optimal pH and pH stability of the recombinant *Cm*Chi1. The optimal pH was determined in 50 mM solutions of various buffers within the pH range 3.5–10.0 (black line). To determine pH stability, the enzyme was incubated at 45 °C for 2 h with various pH buffers (gray line), and the residual activities were measured. **b** Optimal temperature and thermal stability of the recombinant *Cm*Chi1. The temperature optimum was determined at different temperatures (25–60 °C) in 50 mM sodium citrate (pH 5.2) (solid squares). To determine the thermostability, the residual activity was measured in 50 mM sodium citrate (pH 5.2) after the enzyme was treated for 2 h at different temperatures (open triangles)

The temperature and pH profiles of chitinase activity are shown in Fig. 5. *Cm*Chi1 showed high levels of activity at pH 4.6–8.5, with an optimum pH of 5.2. Little activity was detected at a pH below 4.2 or above 9.6, but *Cm*Chi1 retained more than 50% of its activity after storage at pH 5.2–8.2 for 2 h (Fig. 5a). *Cm*Chi1 was active at 25–60 °C, with the highest activity at 50 °C. *Cm*Chi1 was stable and retained > 95% activity for 2 h at temperatures of < 45 °C, but it was unstable at temperatures > 50 °C (Fig. 5b). These results suggested that *Cm*Chi1 is a mesophilic and acidic enzyme.

The optimum pH of reported chitinases (ChiC, ChiD, ChiE and ChiG) from *C. shinanonensis* is 5.0 with optimum temperatures ranging from 40 to 45 °C [18], which is similar with that of *Cm*Chi1 (50 °C and pH 5.2). However, the optimum pHs of chitinases from *Microbispora* sp. V2 (pH 3.0) [37], *P. thermoaerophilus* TC22-2b (4.0) [38], *P. barengoltzii* (pH 4.5), *Chitinibacter* sp. GC72 (pH 6.8) [19] and *Paenibacillus pasadenensis* NCIM 5434 (pH 10.0) [39] were different from that of *Cm*Chi1. In addition, the temperature and pH stabilities were better than those of the chitinases from other bacteria, such as the chitinases from *Halobacterium salinarum* and *Chitinibacter* sp. GC72 [19].

The effects of metal ions on *Cm*Chi1 activity were investigated in this study. All counter-ions of the used metal ions were Cl^−^. EDTA did not inhibit the enzymatic activity at a final concentration of 10 mM, which indicates *Cm*Chi1 is non-metal dependent (Table 3). The activity was completely inhibited by Al^3+^ and strongly inhibited by Ag^+^, Cu^2+^, Fe^2+^. Zn^2+^, K^+^, Ba^2+^ and Na^+^ had a strengthening effect on *Cm*Chi1 activity. Vaidya et al. also reported a chitinase from *Alcaligenes xylosoxidans*, which could be inhibited by 25% in the presence of 5 mM Cu^2+^ or Na^+^ [40]. The chitinase from *Serratia plymuthica* was stimulated by 120, 150 and 240% in the presence of Ca^2+^, Co^2+^ or Mn^2+^ and inhibited by 80% in the presence of Cu^2+^ at a concentration of 10 mM [41]. However, the activity of *Cm*Chi1 was slightly inhibited at the exist of the Mn^2+^.

**Table 3:** Effects of metal ions on the activity of *Cm*Chi1

Metal ion	Chemical	Concentration (mM)	Relative activity (%)
No addition	None	0	100
Ca^2+^	CaCl_2_	10	94.4 ± 3.0
Co^2+^	CoCl_2_	10	62.2 ± 1.4
K^+^	KCl	10	115.4 ± 2.1
Cu^2+^	CuCl_2_·2H_2_O	10	17.3 ± 0.8
Mg^2+^	MgCl_2_	10	90.5 ± 2.6
Zn^2+^	ZnCl_2_	10	45.3 ± 1.1
Al^3+^	AlCl_3_	10	0
Mn^2+^	MnCl_2_	10	80.6 ± 2.9
Ag^+^	AgCl	10	16.7 ± 0.9
Ba^2+^	BaCl_2_	10	115.7 ± 0.2
Fe^2+^	FeCl_2_	10	31.6 ± 1.0
Na^+^	NaCl	10	113.7 ± 1.8
Ni^2+^	NiCl_2_	10	61.5 ± 2
EDTA	EDTA	10	102.1 ± 2.7

### Substrate specificity of *Cm*Chi1

The ability of *Cm*Chi1 to hydrolyze various substrates was investigated under standard conditions. Of the substrates tested, CC was most effectively hydrolyzed by the enzyme with an activity of 15.3 U/mg. In addition, *Cm*Chi1 displayed low activities toward powdery chitin (1.1 U/mg) and chitosan (0.3 U/mg). No activity was observed for CMC, hemicellulose and amylose (Table 4). These phenomena showed that *Cm*Chi1 exhibits strict substrate specificity, which is similar to chitinase *Pb*Chi70 from *P. barengoltzii* CAU904 [28], which shows high activity toward colloidal chitin (30.1 U/mg), trace activity toward powdered chitin (0.5 U/mg) and no activity toward CMC. The reaction of *Cm*Chi1 with various chitin oligosaccharides was also tested. *Cm*Chi1 showed high activities for (GlcNAc)_6_ (121.4 U/mg), (GlcNAc)_5_ (180.7 U/mg), (GlcNAc)_4_ (83.3 U/mg), (GlcNAc)_3_ (38.4 U/mg) and *p*-NP-(GlcNAc)_2_ (27.3 U/mg).

**Table 4:** Substrate specificity of *Cm*Chi1

Substrate	Specific activity (U/mg)
CC	15.3 ± 0.3
Chitin powder	1.1 ± 0.05
CMC	0
Hemicellulose	0
Amylose	0
Chitosan^a^	0.3 ± 0.02
*p*-NP-GlcNAc	0.2 ± 0.01
*p*-NP-(GlcNAc)_2_	27.3 ± 0.6
(GlcNAc)_2_	0.5 ± 0.03
(GlcNAc)_3_	38.4 ± 0.8
(GlcNAc)_4_	83.3 ± 3.1
(GlcNAc)_5_	180.7 ± 1.6
(GlcNAc)_6_	121.4 ± 1.7

^a^The degree of deacetylation of chitosan used was 85%

Furthermore, the kinetic parameters of *Cm*Chi1 toward CC and *p*-NP-(GlcNAc) _2_ were investigated (Table 5). The [s]-velocity plots of CC and *p*-NP-(GlcNAc) _2_ were shown in Additional file 1: Figure S1, Additional file 2: Figure S2, respectively. The *K*_m_, *k*_cat_ and *k*_cat_/*K*_m_ values were determined to be 2.4 ± 0.12 mg/mL, 18.6 ± 0.78 s^−1^ and 7.8 ± 0.11 mL/s/mg for CC, and 0.58 ± 0.04 mg/mL, 42.5 ± 1.4 s^−1^ and 73.5 ± 2.6 mL/s/μmol for *p*-NP-(GlcNAc)_2_, respectively.

**Table 5:** Kinetic parameters of *Cm*Chi1

Substrate	CC	*p*-NP-(GlcNAc)_2_
*V*_max_	526.2 ± 22.2 (μmol/min/L)	108.6 ± 3.3 (μmol/min/L)
*K*_m_	2.4 ± 0.12 (mg/mL)	0.58 ± 0.04 (μmol/mL)
*k*_cat_	18.6 ± 0.78 (s^−1^)	42.5 ± 1.4 (s^−1^)
*k*_cat_/*K*_m_	7.8 ± 0.11 (mL/s/mg)	73.5 ± 2.6 (mL/s/μmol)

### Hydrolysis mechanism of *Cm*Chi1

**Fig. 6:**
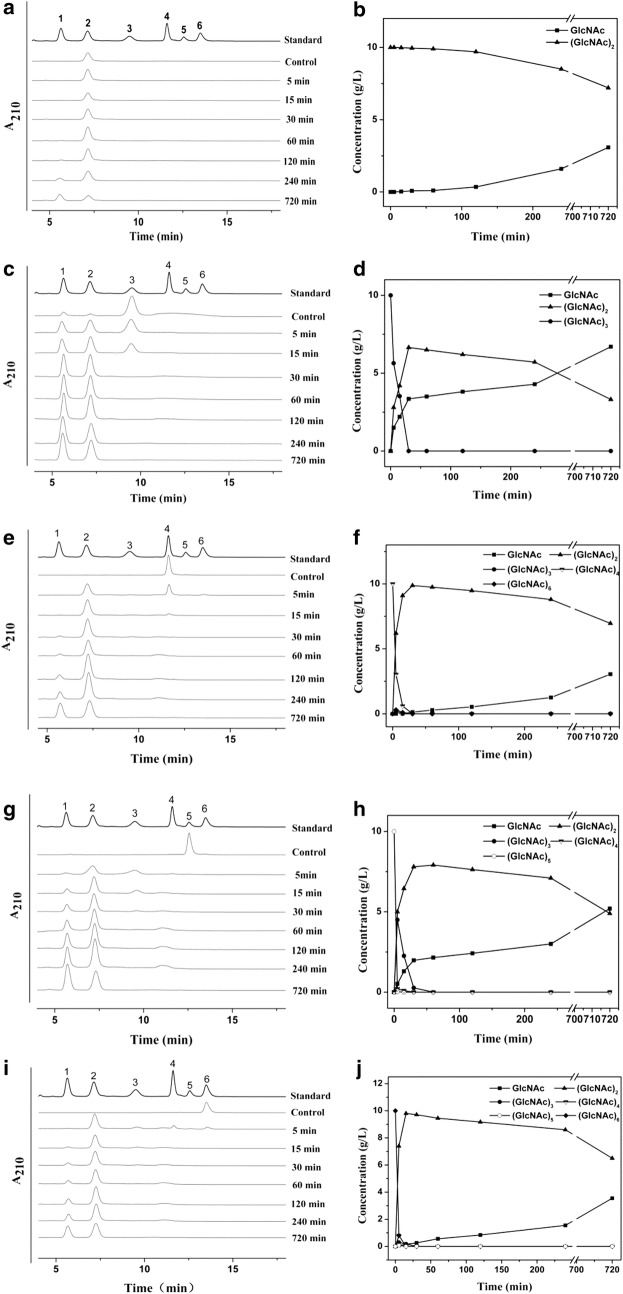
Cleavage pattern of (GlcNAc)_2–6_ by *Cm*Chi1. Numbers 1–6 represent GlcNAc to (GlcNAc)_6_. **a**, **c**, **e**, **g**, **i** show the hydrolysis products from (GlcNAc)_2–6_ using HPLC, respectively. **b**, **d**, **f**, **h**, **j** show the concentrations of CHOS products generated during the reaction time course from (GlcNAc)_2–6_, respectively

To evaluate the hydrolysis mechanism of *Cm*Chi1, *N*-acetyl-CHOS (DP 2–6) and CC were used as substrates for hydrolysis. *Cm*Chi1 rapidly hydrolyzed (GlcNAc)_3–6_ and slowly hydrolyzed (GlcNAc)_2_ (Fig. 6). *Cm*Chi1 rapidly released (GlcNAc)_2_ as the main product from (GlcNAc)_3_, (GlcNAc)_4_, (GlcNAc)_5_, (GlcNAc)_6_ and CC, which suggests *Cm*Chi1 is an exochitinase. Meanwhile, GlcNAc was released from (GlcNAc)_2,_ which showed *Cm*Chi1 also possesses *N*-acetyl-β-d-glucosaminidase activity. However, no GlcNAc was released from (GlcNAc)_2_ within 30 min, which showed the *N*-acetyl-β-d-glucosaminidase activity of *Cm*Chi1 is poor. However, minor (GlcNAc)_3_, (GlcNAc)_4_ and (GlcNAc)_3–5_ were each released from (GlcNAc)_4_, (GlcNAc)_5_ and (GlcNAc)_6_ in 5 min, respectively, which showed that these cleavages were not achieved by *N*-acetyl-β-d-glucosaminidase activity. Further, minor (GlcNAc)_3–4_ and much GlcNAc were detected in the CC hydrolysis before 15 min and after 30 min, which suggested that *Cm*Chi1 has some endo cleavage activity. The reaction mechanism involves oligomers of GlcNAc and dimers generated from chitin by endo activity and exo activity, respectively. Meanwhile, exo activity cleaves the odd oligomers of GlcNAc to GlcNAc and (GlcNAc)_2_.

The hydrolysis profile of *Cm*Chi1 is different from that of most chitinases from *T. kodakaraensis* KOD1 [42], *Enterobacter cloacae* subsp. *cloacae* [43] and *P. barengoltzii* [12]. For instance, Fu et al. reported an exochitinase *Pb*Chi74 from *P. barengoltzii* possesses both exo activity and *N*-acetyl-β-d-glucosaminidase activity but no endo activity [12]. Yang et al. reported chitinase *Pb*Chi70 from *P. barengoltzii* only has exo activity [28].

In conclusion, these phenomena suggest *Cm*Chi1 may be a novel multi-functional chitinase, which has both exo and endo activities with *N*-acetyl-β-d-glucosaminidase activity. Moreover, the exo activity outweighs the *N*-acetyl-β-d-glucosaminidase and endo activities, which leads to the shorter duration of (GlcNAc)_3–6_ than for other reported chitinases [30], which all demonstrated (GlcNAc)_3–6_ accumulation over a long time period.

### GlcNAc production and separation from CC by *Cm*Chi1

**Fig. 7:**
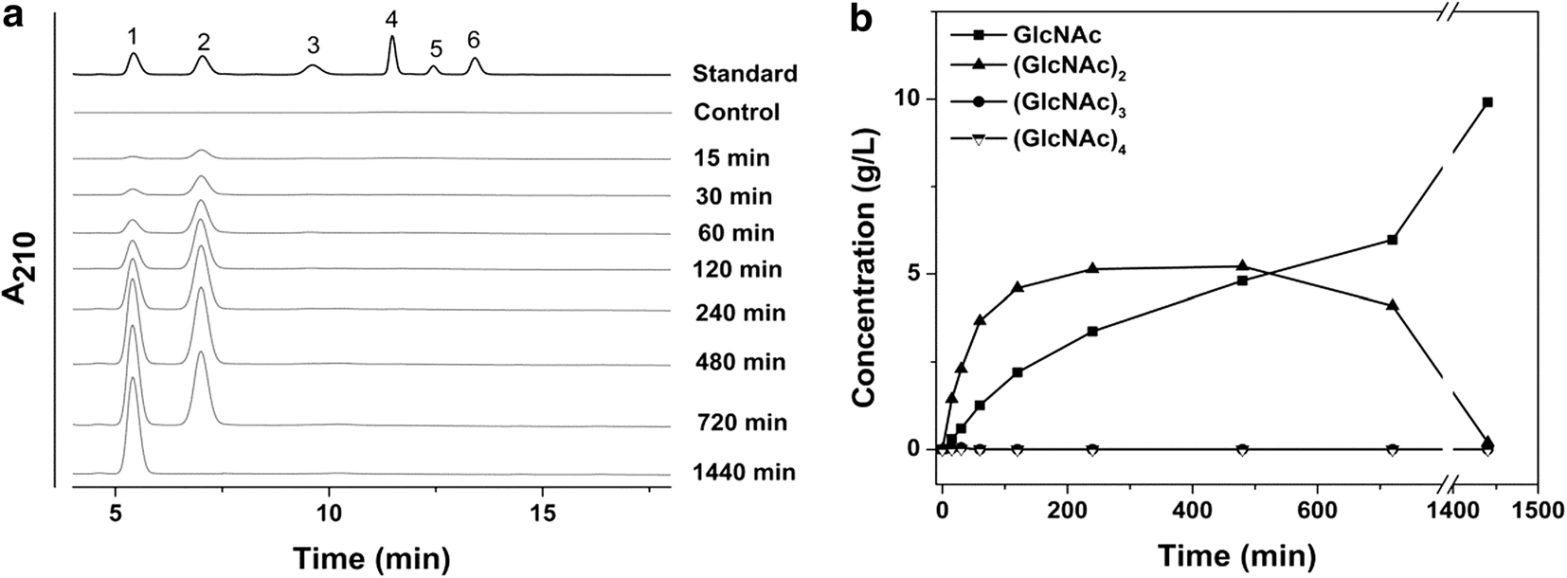
Hydrolysis of CC by *Cm*Chi1. The reactions contained 50 μg *Cm*Chi1 and 1% (w/v) CC, and were performed in sodium citrate buffer (pH 5.2) at 50 °C, and aliquots were withdrawn at different time intervals and analyzed by HPLC. Numbers 1–6 represent GlcNAc to (GlcNAc)_6_. **a** HPLC profiles of reaction products from CC. **b** The time courses of products generated from CC. Products were quantified from the respective areas using standard curves of (GlcNAc)_1–6_

GlcNAc batch production from CC by *Cm*Chi1 was investigated, as shown in Fig. 7. The concentrations of GlcNAc and (GlcNAc)_2_ increased over time until the CC was completely hydrolyzed, and then (GlcNAc)_2_ was converted to GlcNAc gradually. Finally, 9.8 g/L of GlcNAc without oligomer was obtained from CC with a 98% yield after 24 h. Yang et al. reported an exochitinase (PbChi70) from *P. barengoltzii*, which mainly hydrolyzed CC to (GlcNAc)_2_ but could not further convert (GlcNAc)_2_ to GlcNAc [28]. Fu et al. also reported hydrolysis of CC to produce GlcNAc using chitinase (PbChi74), which possesses both exo activity and *N*-acetyl-β-d-glucosaminidase activity. But (GlcNAc)_2_ was always present during the process and could not be converted to GlcNAc completely. In their study, extra *N*-acetyl-β-d-glucosaminidase was added to enhance the yield of GlcNAc, and they finally obtained 27.8 mg/mL of GlcNAc with a conversion ratio of 92.6% [12]. Our results demonstrated the *Cm*Chi1 can achieve GlcNAc production without the assistance of other enzymes and has the potential to be scaled up for industrial production.

Moreover, the main advantage of the use of the purified multi-functional chitinase is that the GlcNAc produced can be separated simply. HPLC analysis showed only one peak at 5.5 min (Additional file 3: Figure S3), revealing the product purity was more than 98% after determination and comparison with the (GlcNAc)_1–6_ standard HPLC curve. The mass spectrum of purified GlcNAc showed three molecular ions at m/z values of 244.0801, 260.0538 and 465.1705, which correspond to GlcNAc (221.0899 Da) with a sodium adduct (22.9898 Da) and a potassium adduct (38.9639 Da), respectively, and two GlcNAc molecules (442.1798 Da) with a sodium adduct (Additional file 4: Figure S4).

## Conclusion

A novel chitinase, *Cm*Chi1, was identified from *Chitinolyticbacter meiyuanensis* SYBC-H1. The *Cm*Chi1 contains a glycosyl hydrolase family 18 (GH18) catalytic module and exhibits no similarity with previously characterized chitinases. *Cm*Chi1 can be purified with a recovery yield of 89% and a specific activity of 15.3 U/mg by colloidal chitin affinity chromatography. Analysis of the hydrolysis products revealed that *Cm*Chi1 exhibits exo-acting, endo-acting and *N*-acetyl-β-d-glucosaminidase activities toward *N*-acetyl CHOS and colloidal chitin (CC) substrates, behavior that makes it different from typical reported chitinases. As a result, *N*-acetyl-d-glucosamine could be produced via hydrolysis of CC using *Cm*Chi1 alone. The physicochemical properties of *Cm*Chi1 suggest that it has potential for commercial development in GlcNAc enzymatic production.

## Additional files

**Additional file 1: Figure S1.** Determination of *K*_m_ and *V*_m_ of the *Cm*Chi1 using CC as the substrate.

**Additional file 2: Figure S2.** Determination of *K*_m_ and *V*_m_ of the *Cm*Chi1 using *p*-NP-(GlcNAc)_2_ as the substrate.

**Additional file 3: Figure S3.** HPLC profile of the GlcNAc product. Numbers 1 to 6 represent GlcNAc to (GlcNAc)_6_. (a): standard samples; (b): product.

**Additional file 4: Figure S4.** MS of profile of the GlcNAc product.
