# Supplementary material for: Molecular characterization of a novel chitinase CmChi1 from Chitinolyticbacter meiyuanensis SYBC-H1 and its use in N-acetyl-d-glucosamine production
Source: Biotechnol Biofuels. 2018 Jun 26;11:179. doi: 10.1186/s13068-018-1169-x (PMC6020246; doi:10.1186/s13068-018-1169-x)
Supplement: Supplementary file 1 — Additional file 1: Figure S1. Determination of Km and Vm of the CmChi1 using CC as the substrate. [file 13068_2018_1169_MOESM1_ESM.doc]

Additional Information for

Molecular characterization of a novel chitinase *Cm*Chi1 from Chitinolyticbacter meiyuanensis SYBC-H1 and its use in N-acetyl-D-glucosamine production

Alei Zhang^a^, Yumei He^a^，Guoguang Wei^a^, Jie Zhou^a,b^, Weiliang Dong^a,b^, Kequan Chen^a,b*^, Pingkai Ouyang^a,b^

*^a^College of Biotechnology and Pharmaceutical Engineering, NanjingTech University, Nanjing, 211800, P.R.China*

*^b^State Key Laboratory of Materials-Oriented Chemical Engineering, NanjingTech University, Nanjing, 211800, P.R.China*

*Corresponding author: Tel.: +86-138-1418-0652

E-mail address: [kqchen@njtech.edu.cn](mailto:kqchen@njtech.edu.cn)

Additional data 1.


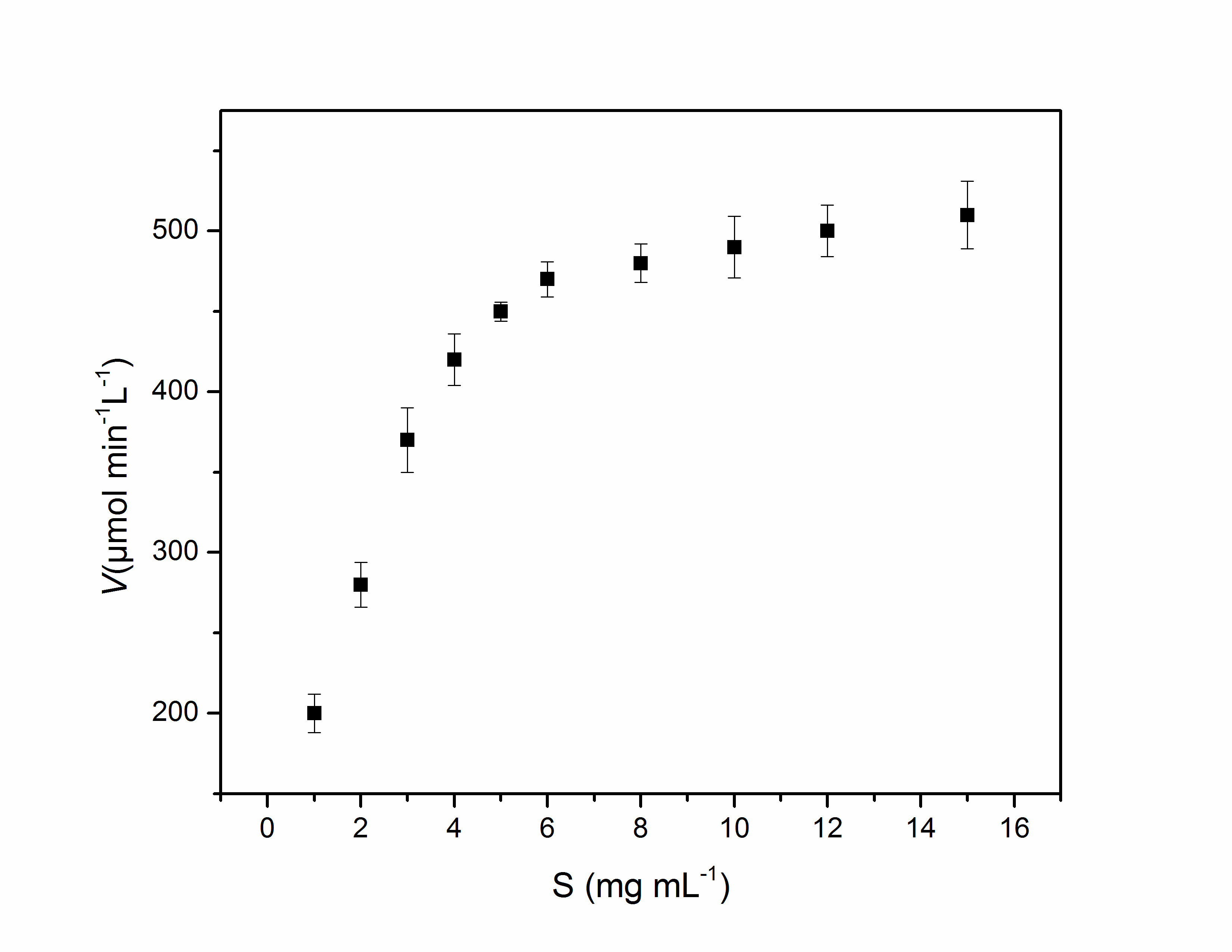


**Figure S1** Determination of *K*_m_ and *V*_m_ of the *Cm*Chi1 using CC as the substrate.
