# Supplementary material for: Molecular characterization of a novel chitinase CmChi1 from Chitinolyticbacter meiyuanensis SYBC-H1 and its use in N-acetyl-d-glucosamine production
Source: Biotechnol Biofuels. 2018 Jun 26;11:179. doi: 10.1186/s13068-018-1169-x (PMC6020246; doi:10.1186/s13068-018-1169-x)
Supplement: Supplementary file 2 — Additional file 2: Figure S2. Determination of Km and Vm of the CmChi1 using p-NP-(GlcNAc)2 as the substrate. [file 13068_2018_1169_MOESM2_ESM.docx]

Additional data 2


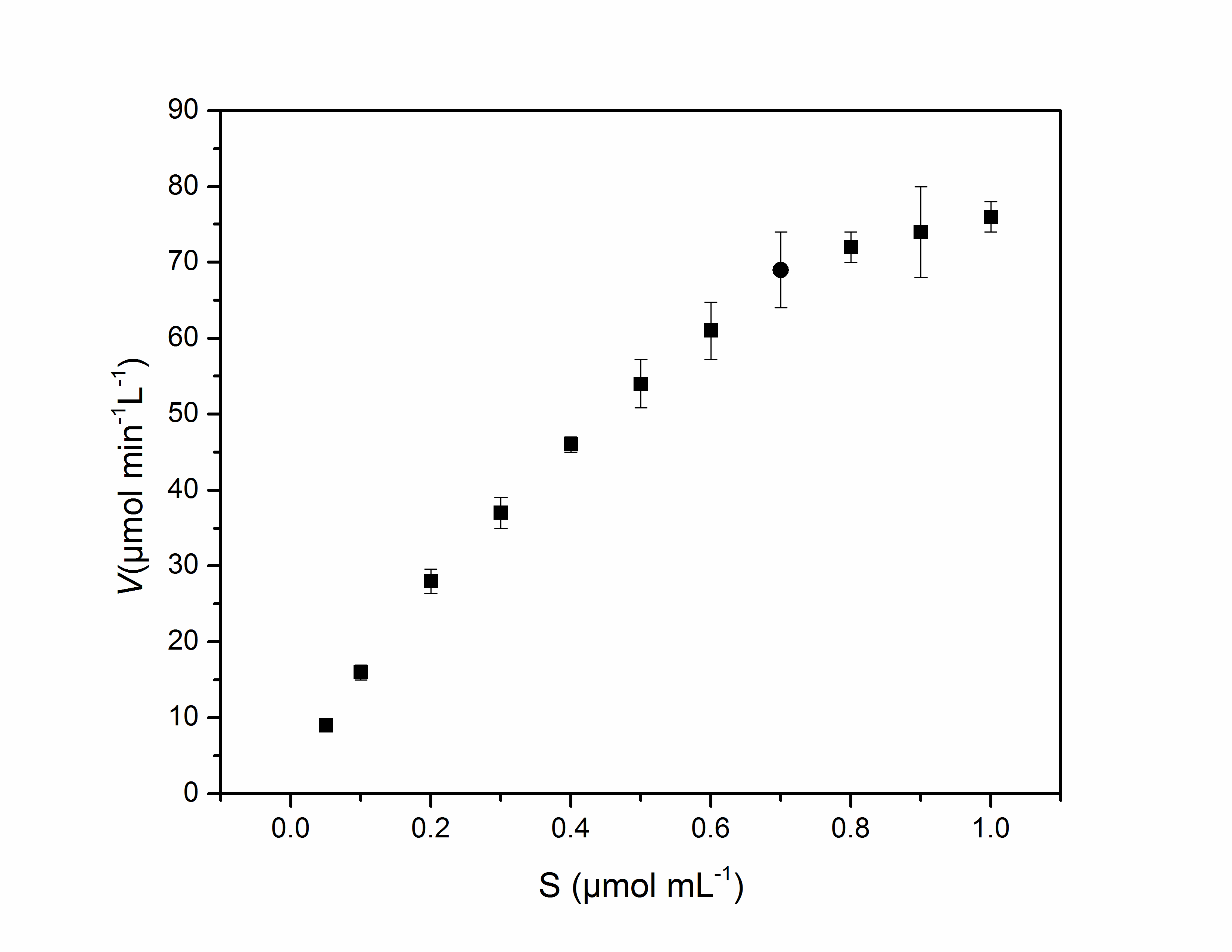


**Figure S2** Determination of *K*_m_ and *V*_m_ of the *Cm*Chi1 using *p*-NP-(GlcNAc)_2_ as the substrate.
