# Supplementary material for: Molecular characterization of a novel chitinase CmChi1 from Chitinolyticbacter meiyuanensis SYBC-H1 and its use in N-acetyl-d-glucosamine production
Source: Biotechnol Biofuels. 2018 Jun 26;11:179. doi: 10.1186/s13068-018-1169-x (PMC6020246; doi:10.1186/s13068-018-1169-x)
Supplement: Supplementary file 3 — Additional file 3: Figure S3. HPLC profile of the GlcNAc product. Numbers 1 to 6 represent GlcNAc to (GlcNAc)6. (a): standard samples; (b): product. [file 13068_2018_1169_MOESM3_ESM.docx]

Additional data 3.

**Figure S3** HPLC profile of the GlcNAc product. Numbers 1 to 6 represent GlcNAc to (GlcNAc)_6_. (a): standard samples; (b): product
