# Supplementary material for: Molecular characterization of a novel chitinase CmChi1 from Chitinolyticbacter meiyuanensis SYBC-H1 and its use in N-acetyl-d-glucosamine production
Source: Biotechnol Biofuels. 2018 Jun 26;11:179. doi: 10.1186/s13068-018-1169-x (PMC6020246; doi:10.1186/s13068-018-1169-x)
Supplement: Supplementary file 4 — Additional file 4: Figure S4. MS of profile of the GlcNAc product. [file 13068_2018_1169_MOESM4_ESM.docx]

Additional data 4.

**Figure S4** MS of profile of the GlcNAc product
